# Habitual Fish Oil Supplementation and Incident Chronic Kidney Disease in the UK Biobank

**DOI:** 10.3390/nu15010022

**Published:** 2022-12-21

**Authors:** Mengyi Liu, Ziliang Ye, Sisi Yang, Yanjun Zhang, Qimeng Wu, Chun Zhou, Panpan He, Yuanyuan Zhang, Fanfan Hou, Xianhui Qin

**Affiliations:** Division of Nephrology, Nanfang Hospital, Southern Medical University, National Clinical Research Center for Kidney Disease, State Key Laboratory of Organ Failure Research, Guangdong Provincial Institute of Nephrology, Guangdong Provincial Key Laboratory of Renal Failure Research, Guangzhou 510515, China

**Keywords:** fish oil, oily fish, nonoily fish, omega-3 polyunsaturated fatty acid, chronic kidney diseases

## Abstract

Background: To explore the relation of habitual fish oil use with the risk of chronic kidney diseases (CKD). Methods: 408,023 participants (54.2% female) without prior CKD and with completed information regarding their consumption of major food groups and fish oil in the UK Biobank were enrolled. Fish oil use and dietary intakes were assessed by touch screen questionnaire and food frequency questionnaire, respectively. Incident CKD was recorded from hospital inpatient records. Results: At baseline, 128,843 (31.6%) participants reported taking fish oil supplements. During a median follow-up period of 12.0 years, a total of 10,782 (2.6%) participants developed CKD. With adjustments for important confounders, habitual fish oil use was associated with a significantly lower hazard of incident CKD (hazard ratio [HR], 0.90; 95% confidence interval [CI], 0.87–0.95), compared with non-use. Consistently, participants reporting ≥2 servings/week of oily fish (HR, 0.86; 95% CI, 0.79–0.94) and nonoily fish (HR, 0.86; 95% CI, 0.77–0.97) consumption had a lower hazard of incident CKD compared to those reporting no consumption ever. Additionally, among the 97,914 participants with data on plasma fatty acid, there were significant inverse relationships of plasma omega-3 polyunsaturated fatty acid (PUFA) (per SD increment, HR, 0.89, 95% CI, 0.84–0.94) and eicosatetraenoic acid (per SD increment, HR, 0.91, 95% CI, 0.87–0.96) with incident CKD. Conclusions: Habitual fish oil use was associated with a lower hazard of CKD, which was further confirmed by the consistent inverse relations between fish consumption and circulating omega-3 PUFA concentration with incident CKD.

## 1. Introduction

Chronic kidney disease (CKD) was associated with higher hazards of renal failure, cardiovascular disease (CVD), and all-cause mortality, and has become a global health and socio-economic burden [1,2,3]. The global age-standardized prevalence of CKD was 10.4% in men and 11.8% in women, and the prevalence is increasing along with the global aging trend and the growing population with obesity [4]. Therefore, understanding more modifiable risk factors for CKD, such as lifestyles and diets, in a community setting is of great clinical and public health significance for establishing primary preventive measures.

Omega-3 polyunsaturated fatty acid (PUFA), including docosahexaenoic acid (DHA) and eicosatetraenoic acid (EPA), has been shown to have a potential role in reducing serum lipids, blood pressure, and inflammation levels, as well as protecting endothelial function, and may therefore be associated with improved renal function [5,6,7]. Accordingly, a few epidemiological studies have reported that lower dietary long-chain omega-3 PUFA [8] intake or lower plasma PUFA concentrations [9] were associated with a higher hazard of incident CKD or a greater decline in creatinine clearance. Nevertheless, previous randomized clinical trials [10,11,12,13] observed no or only a small beneficial effect of omega-3 PUFA on the decline of the glomerular filtration rate (GFR), and did not observe a significant benefit in the reducing risk of CKD. Of note, these trials mainly evaluated the effect of omega-3 PUFA supplementation in high-risk populations (e.g., patients with diabetes or myocardial infarction, etc.), and the small sample sizes (less than 2500 for all studies) may have made these trials underpowered to show any statistically significant benefit on CKD incidence. Therefore, large-scale cohort studies with long-term follow-up are needed to examine the effectiveness of fish oil supplements in the general population, especially in real-life settings, to generalize the findings to more inclusive populations [14].

As such, our current study aimed at evaluating the associations of habitual fish oil use, as well as consumption of oily and nonoily fish and plasma omega-3 PUFA concentration, with the hazard of incident CKD using data from the UK Biobank study, a population-based cohort of nearly half a million general adults.

## 2. Methods

### 2.1. Data Source and Study Population

As previously described [15,16], the UK Biobank is a large, observational, population-based cohort recruiting half a million adult residents of the UK, aged 37–73 years, from 1 of 22 assessment centers across the United Kingdom (England, Wales, and Scotland) between 2006 and 2010. Participants were asked to complete comprehensive questionnaires assessing sociodemographic, lifestyle, and health related information, receive physical examinations, and provide biological samples. The UK Biobank was approved by the North West Research Ethics Committee (06/MRE08/65) and all participants signed an informed consent document.

In this study, we included participants who had complete information on kidney function, fish intake, fish oil use, and important covariates. Supplementary Materials Table S1 demonstrated the characteristics of the included (*n* = 439,542) and excluded (*n* = 62,872) persons. Of the 439,542 participants, those with prior CKD (*n* = 31,519) were further excluded. As such, 408,023 participants were enrolled in the current analysis (Supplementary Materials Figure S1).

### 2.2. Ascertainment of Exposure

At baseline, habitual fish oil use was recorded using an electronic questionnaire. Participants were asked the following question: “Do you regularly take any of the following?” They could then select their answer from a list of supplements, including fish oil. Data regarding habitual dietary intake were obtained from a standardized and validated touchscreen food frequency questionnaire (FFQ) that included 29 questions about the average consumption of major foods or food groups in the last year [17]. Intakes of oily and nonoily fish were assessed by asking how often (“never”, “less than once a week”, “once a week”, “2–4 times a week”, “5–6 times a week”, or “once or more daily”) they consumed oily fish (e.g., salmon anchovies, trout swordfish, mackerel bloater, herring cacha, sardines carp, pilchards hilsa, kipper jack fish, eel katla, whitebait orange roughy, tuna [fresh only] pangas, sprats) and other types of fish (e.g., cod, tinned tuna, haddock).

Approximate 120,000 EDTA plasma samples were randomly selected from the blood samples collected at baseline. Plasma concentrations of fatty acids and fatty acid compositions were determined using a high-throughput NMR-based metabolic biomarker profiling platform developed by Nightingale Health Ltd, which was known for its absence of batch effects and high repeatability over time. More details about the biomarker measurements and quality control can be found online (https://biobank.ctsu.ox.ac.uk/crystal/label.cgi?id=220; accessed on 17 December 2022).

### 2.3. Ascertainment of Covariates

Lifestyle and demographic information, including age, sex, race, Townsend Deprivation Index (TDI), smoking status, alcohol consumption, vitamin or mineral supplementations, and comorbidities (hypertension, diabetes, and high cholesterol) was available through several touch-screen computer-based questionnaires. Prevalent hypertension was defined as a self-reported history of hypertension, being under antihypertensive treatment, a systolic blood pressure ≥140 mmHg, or a diastolic blood pressure ≥90 mmHg. Prevalent diabetes was identified by considering the type and diagnosis sources of diabetes [18]. A healthy diet score was calculated based on five dietary goals for ideal cardiovascular health: fruit intake ≥4.5 pieces/day, vegetable intake ≥4.5 servings/week, fish intake ≥2 servings/week, processed meat intake < twice/week, and unprocessed red meat intake ≤5 times/week [19], ranging from 0 to 5. One point was given for each favorable diet factor, with a higher score indicating a healthier diet [19]. Serum and urinary creatinine were performed at a dedicated central laboratory, and eGFR was calculated by CKD-EPI equation [20].

### 2.4. Study Outcome

The study outcome was incident CKD. The date of CKD was identified chiefly by using linkage with hospital admission data, and outcomes were defined according to the code of International Classification of Diseases (ICD) edition 9, ICD edition 10, and the Office of Population Censuses and Surveys Classification of Interventions and Procedures, version 4 (OPCS-4) (Supplementary Materials Table S2). The follow-up person*–*year for each participant was calculated from baseline until the date of death, first date of CKD diagnosis, date of loss to follow-up, or end of follow-up, whichever came first.

### 2.5. Statistical Analysis

Baseline characteristics were summarized across fish oil use (yes or no) as mean ± standard deviation (SD) and proportions for continuous and categorical variables, respectively, and compared using chi-square tests and t tests for categorical and continuous variables, accordingly.

The relation between habitual fish oil use and incident CKD was estimated by Cox proportional hazards models, and hazard ratio (HR) and 95% confidence interval (CI) of CKD was calculated. The interaction between exposures and follow-up time was used to evaluate the proportional hazard assumption, and no violation was detected. Covariates that were known to be traditional or suspected risk factors for CKD, including age, sex, race, TDI, BMI, smoking status, alcohol consumption, diabetes, hypertension, high cholesterol, vitamin or mineral supplementation, healthy diet score, and renal function (eGFR and UACR), were considered as potential confounders and were adjusted for in multivariable models. We further adjusted for oily and nonoily fish intakes and CKD genetic risk scores (GRS), which was created using the R package *bigsnpr* with 263 single nucleotide polymorphisms that showed significant associations with kidney function [21], to control the potential effect of fish consumption and genetic susceptibility.

In order to verify the relation between omega-3 PUFA and CKD incidence, the association of oily/nonoily fish intake, plasma omega-3 PUFA, and DHA with incident CKD were also evaluated by using a similar strategy. For fish intake, fish oil supplement was further adjusted and oily/nonoily fish intake was mutually adjusted in a multivariable model. For plasma omega-3 PUFA and DHA, analysis was performed in 97,914 participants with data on plasma fatty acid, and fish oil supplement and plasma saturated fatty acid, monounsaturated fatty acid, and omega-6 PUFA were further adjusted in multivariable model. 

In order to explore possible modifiers of fish oil supplement with CKD, stratified analysis was conducted and interaction terms were added to the Cox models to explore the potential effect modifications.

A two-tailed *p* < 0.05 was considered to be statistically significant in all analyses. Analyses were performed using R 4.1.1 software (R Foundation for Statistical Computing, Vienna, Austria, http://www.R-project.org, accessed on 17 December 2022).

## 3. Results

### 3.1. Study Participants and Characteristics

Among the 408,023 participants included in the present study, 186,931 (45.8%) were male, with a mean age of 56.3 years. Overall, 128,843 (31.6%) participants reported taking fish oil at baseline. The characteristics of study participants were shown by fish oil use in Table 1. Compared with fish oil non-users, fish oil users were older, more likely to be female, White, non-current smokers, vitamin or mineral supplement users, and tended to have a lower BMI, lower TDI, higher alcohol intake, healthy diet, higher prevalence of hypertension, and high cholesterol, but a lower prevalence of diabetes. The characteristics of the population according to categories of fish consumption and quintiles of plasma omega-3 PUFA were also shown in Supplementary Materials Tables S3 and S4, respectively.

### 3.2. Fish Oil Supplements and Incident CKD

**Figure 1:**
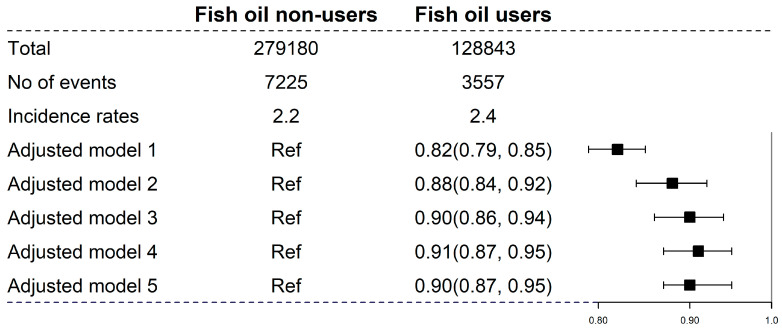
Relation of fish oil supplement with risk of incident chronic kidney disease. Incidence rates were expressed as per 1000 person*–*years, and “Ref” means the reference group with an HR of 1.00. Adjusted Model 1: Adjusted for age, and sex; Adjusted Model 2: adjusted for the covariates in Model 1 and further adjusted for race, Townsend Deprivation Index, body mass index, smoking status, alcohol consumption, diabetes, hypertension, high cholesterol, vitamin or mineral supplementation, and healthy diet score; Adjusted Model 3: adjusted for the covariates in Model 2 and further adjusted for renal function (estimated glomerular filtration rate, and urine albumin: creatinine ratio); Adjusted Model 4: adjusted for the covariates in Model 3 and further adjusted for oily fish and nonoily fish consumption; Adjusted Model 5: adjusted for the covariates in Model 4 and further adjusted for CKD genetic risk score.

A total of 10,782 (2.6%) participants developed CKD over a median follow-up period of 12.0 years (4,763,189 person*–*years). Compared with fish oil non-users, fish oil users had a lower hazard of CKD (HR, 0.88, 95% CI, 0.84–0.92; Figure 1). Further adjustments of renal function at baseline, oily and nonoily fish consumption, and CKD GRS did not substantially change the results (Figure 1).

**Figure 2:**
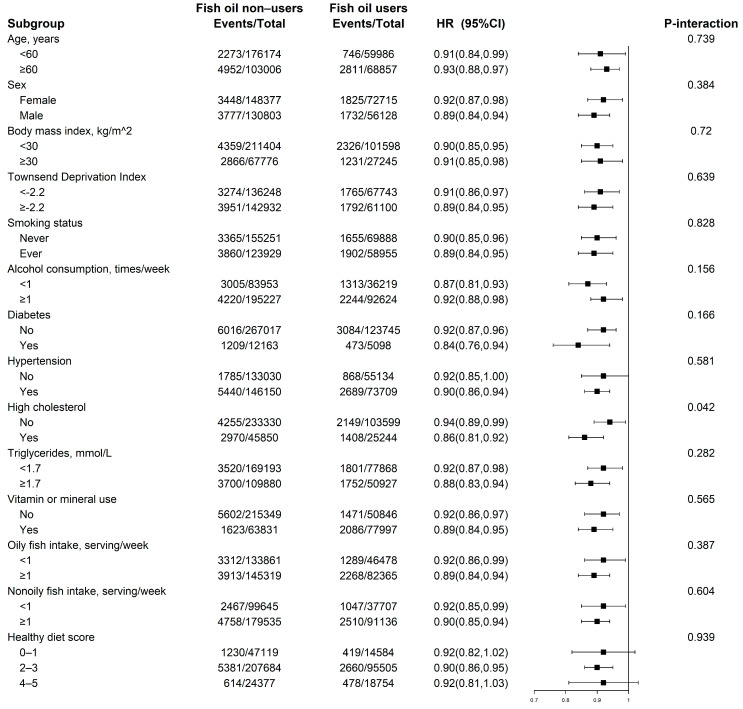
Association of fish oil supplement use and the risk of chronic kidney disease stratified by potential risk factors. Results were adjusted for age, sex, race, Townsend Deprivation Index, body mass index, smoking status, alcohol consumption, diabetes, hypertension, high cholesterol, vitamin or mineral supplementation, healthy diet score, estimated glomerular filtration rate, urine albumin: creatinine ratio, oily fish intake, and nonoily fish intake, if not already stratified.

In the stratified analyses (Figure 2), the relation of fish oil use with incident CKD was weaker among the participants without prevalent high cholesterol (*P* for interaction = 0.042; Figure 2). No other variables, including age, sex, BMI, TDI, smoking status, alcohol consumption, comorbidities (hypertension and diabetes), triglycerides, vitamin or mineral use, oily fish consumption, nonoily fish consumption, and healthy diet score, showed any significant effect modifications on the relation of fish oil supplements and incident CKD (all *P* for interaction ≥ 0.05; Figure 2).

### 3.3. Oily and Nonoily Fish Consumption with Incident CKD

A total of 73,063 (17.9%) and 66,868 (16.4%) participants reported taking ≥2 servings/week of oily fish and nonoily fish at baseline, respectively. Overall, there was a significant inverse relation between oily fish consumption and the hazard of CKD (per 1 serving/week; HR, 0.97, 95% CI, 0.95–0.99). Participants who consumed ≥2 servings/week of oily fish had a 14% (95% CI, 6–21%) lower hazard of CKD compared to those who did not consume oily fish (Table 2). Meanwhile, there was also a relatively weaker inverse relation between nonoily fish intake and incident CKD (per 1 serving/week; HR, 0.98, 95% CI, 0.96–1.00) (Table 2).

### 3.4. Plasma Omega-3 PUFA and DHA with Incident CKD

Among the 97,914 participants with data on plasma fatty acid, 2583 (2.6%) participants developed incident CKD over a median follow-up period of 12.0 years (1,145,893 person*–*years).

Overall, there were significant inverse relationships of plasma omega-3 PUFA (per SD [0.22 mmol/L] increment, HR, 0.89, 95% CI, 0.84–0.94) and DHA (per SD [0.08 mmol/L] increment, HR, 0.91, 95% CI, 0.87–0.96) with incident CKD (Table 3). Accordingly, when plasma omega-3 PUFA and DHA were categorized as quintiles, compared with participants in the first quintile, a significantly lower hazard of incident CKD was observed in those in the second (HR, 0.87; 95% CI: 0.78–0.98), third (HR, 0.82; 95% CI: 0.73–0.93), and fourth (HR, 0.72; 95% CI: 0.63–0.83) quintile of omega-3 PUFA, and those in the second (HR, 0.83; 95% CI: 0.75–0.99), third (HR, 0.79; 95% CI: 0.70–0.89), and fourth (HR, 0.76; 95% CI: 0.67–0.87) quintile of DHA, respectively (Table 3).

## 4. Discussion

In this large-scale longitudinal cohort study in general individuals without prior CKD, habitual fish oil supplementation was associated with a significantly lower hazard of incident CKD, independent of traditional risk factors and CKD GRS. In addition, both intakes of oily and nonoily fish and plasma concentrations of omega-3 PUFA and DHA were significantly and inversely associated with the hazard of incident CKD, all of which suggest that omega-3 PUFA may be beneficial for the primary prevention of CKD.

Several randomized clinical trials have evaluated the effect of supplementation with fish oil on renal function and showed conflicting results. A meta-analysis including 17 small clinical trials with 626 participants with predominantly IgA nephropathy and diabetes (pooled effect size, 0.11; 95% CI: −0.07, 0.29) [13] and a subsequent clinical trial in 1312 diabetics (difference, 0.9 mL/min/1.73 m^2^; 95% CI: −0.7 to 2.6) [12] found that supplementation with omega-3 PUFA did not slow the decline of GFR. Nevertheless, despite the null effect on new-onset CKD and renal function rapid decline, another trial conducted in 2,344 older patients with myocardial infarction (19% with diabetes) reported that supplementation with EPA plus DHA attenuated eGFR decline by 2.1 mL/min/1.73 m^2^ over 3.3 years [11]. A possible explanation for these conflicting results is that some of these previous studies had relatively small samples and short observation periods, thus lacking sufficient statistical power. More importantly, possibly due to the limited sample sizes, the above trials mainly used the changes in GFR as the major study outcome, which is usually relatively weak in clinical significance. To date, only one trial had reported a lower and nonsignificant odds ratio of incident CKD (odds ratio, 0.83; 95% CI, 0.58, 1.18) associated with EPA plus DHA supplementation compared with placebo [11]. Based on a population-based prospective study design with a large sample size and a long follow-up period that improves the accuracy and reliability of the effect estimates, our study provided an opportunity to detect a significantly inverse relation between fish oil supplementation and the hazard of incident CKD in real-life settings. To our knowledge, this is the first and largest study evaluating the relation between fish oil supplementation and incident CKD in the general population.

Of note, evidence from a meta-analysis of three cohorts, including 21,226 participants, did not find a statistically significant relation between fish intake with CKD risk (OR, 0.94; 95%CI: 0.86–1.02) [22]. The relatively small sample size may be the main explanation for the null relation between fish intake and risk of CKD in this meta-analysis. Our present study reported that intakes of both oily and nonoily fish were significantly and inversely related to the risk of incident CKD. Notably, though our study did not show a significant interaction between fish oil supplementation and fish consumption on incident CKD, a slightly stronger inverse association was found in those with a higher level of fish consumption, indicating that even among individuals who already have relatively higher omega-3 PUFA levels through fish intake, supplementation with fish oil containing high amounts of marine omega-3 PUFA may still further reduce the risk of CKD. Moreover, in our current study, the healthy diet score, which was constructed based on five dietary goals, did not significantly modify the association between fish oil supplementation and incident CKD, suggesting that the consumption levels of these foods could not affect the beneficial effect of fish oil supplementation on incident CKD.

In addition, we also found inverse relations between circulating omega-3 PUFA and DHA levels, which could better reflect the bio-availability of omega-3 PUFA consumption, with the hazard of incident CKD. Consistently, a previous study also showed that a higher plasma omega-3 PUFA was associated with a smaller decline in creatinine clearance over three years, although this trend was not statistically significant [9]. Taken together, by considering the fish oil use, dietary oily and nonoily fish intakes, and circulating omega-3 PUFA simultaneously, our findings provided consistent results to support the inverse relation of fish oil use and incident CKD.

Several possible mechanisms could support our findings. Firstly, it has been proposed that omega-3 PUFA decreased serum triglyceride concentrations by lowering triglyceride synthesis, decreasing the incorporation of triglyceride into very-low-density lipoproteins, reducing triglyceride secretion, and enhancing triglyceride clearance from very-low-density lipoproteins particles [23,24]. A previous meta-analysis including 21 clinical trials found that omega-3 PUFA treatment led to a significantly beneficial effect on triglyceride, total cholesterol, non-high-density lipoprotein cholesterol, and very low-density lipoprotein cholesterol [25]. Accordingly, our study observed a stronger inverse relation between fish oil supplementation and CKD among those with prevalent high cholesterol. Secondly, inflammation and oxidative damage were key patho-mechanisms of dysfunction in the kidneys [26]. On the one hand, omega-3 PUFA may reduce the production of inflammatory eicosanoids partly through incorporating in the phospholipids of cell membranes and replacing arachidonic acids as substrates for cyclo-oxygenase and lipoxygenase enzymes and cytokines, and inhibit the production of inflammatory cytokines possibly by producing direct effects on the intracellular signaling pathways that activate transcription factors [5,27], thus protecting kidney function. Moreover, EPA and DHA are also recognized as precursors for the synthesis of novel specialized pro-resolving mediators, including resolvins, protectins, and maresins [27]. On the other hand, omega-3 PUFA has been reported to reduce oxidative stress-related mitochondrial dysfunction and endothelial cell apoptosis via an increased activity of endogenous antioxidant enzymes [28]. Thirdly, omega-3 PUFA could block transforming growth factor β1-induced fibroblast activation, thus having a potentially anti-fibrotic effect [6]. However, more studies are needed to further investigate the underlying mechanisms. This study had several advantages, including a large-scale prospective study design, which provided sufficient statistical power to show the benefit of fish oil supplementation in a real-life setting, and a comprehensive assessment of fish oil supplementation, dietary oily and nonoily fish consumption, and circulating omega-3 PUFA at the same time, to ensure reliability and robustness of the results. However, several limitations should also be noted. First, limited to an observational study, we cannot completely exclude the possibility of residual confounding. Moreover, it is hard to distinguish the effects of a healthy lifestyle from habitual fish oil use. Therefore, although a series of covariates were carefully adjusted for and stratified analysis was performed, a healthy lifestyle or other unmeasured or unknown factors might contribute to the observed relation. Second, we could not explore the dose–response relation and the appropriate supplementation duration of fish oil due to unavailable information on the fish oil use, such as the dose, formulation, frequency, and duration. Third, we could not evaluate the relations for more-specific types of fish, fish preparation methods, or plasma omega-3 PUFA due to the lack of available data. Fourth, we primarily utilized ICD codes to identify outcomes, and thus misclassification of patients with incident CKD remained possible, which might attenuate findings toward the null and underestimate the magnitudes of the true relation. Fifth, the participants were mainly of European descent and healthier than the general adults in the UK [29]; whether the findings can be generalized to other ethnic groups and regions remained unclear. Further studies to verify the results are necessary.

## 5. Conclusions

In conclusion, our study showed that habitual fish oil supplementation, consumption of oily and nonoily fish, and circulating omega-3 PUFA were all inversely associated with the hazard of CKD in general adults, thus supporting the use of fish oil and the current dietary recommendation regarding increasing oily fish intake for CKD prevention. However, additional prospective studies are needed to verify our results in other individuals with different sociodemographic and lifestyle backgrounds.

## Figures and Tables

**Table 1:** Baseline population characteristics by fish oil supplement use *.

	Total	Fish Oil Non-Users	Fish Oil Users	*p*-Value
N	408,023	279,180	128,843	
Age, years	56.3 ± 8.1	55.3 ± 8.1	58.5 ± 7.4	<0.001
Male, No. (%)	186,931 (45.8)	130,803 (46.9)	56,128 (43.6)	<0.001
White, No. (%)	388,982 (95.3)	265,559 (95.1)	123,423 (95.8)	<0.001
TDI	−1.4 ± 3.0	−1.3 ± 3.1	−1.6 ± 2.9	<0.001
BMI, kg/m^2^	27.3 ± 4.7	27.4 ± 4.8	27.1 ± 4.4	<0.001
Smoking status, No. (%)				<0.001
Never	225,139 (55.2)	155,251 (55.6)	69,888 (54.2)	
Former	141,165 (34.6)	92,480 (33.1)	48,685 (37.8)	
Current	41,719 (10.2)	31,449 (11.3)	10,270 (8.0)	
Alcohol consumption, No. (%)				<0.001
Never	30,070 (7.4)	21,392 (7.7)	8678 (6.7)	
<1 times/week	90,102 (22.1)	62,561 (22.4)	27,541 (21.4)	
1–2 times/week	106,302 (26.1)	72,635 (26.0)	33,667 (26.1)	
3–4 times/week	96,943 (23.8)	65,341 (23.4)	31,602 (24.5)	
>4 times/week	84,606 (20.7)	57,251 (20.5)	27,355 (21.2)	
Disease history, No. (%)				
Diabetes	17,261 (4.2)	12,163 (4.4)	5098 (4.0)	<0.001
Hypertension	219,859 (53.9)	146,150 (52.3)	73,709 (57.2)	<0.001
High cholesterol	71,094 (17.4)	45,850 (16.4)	25,244 (19.6)	<0.001
Healthy diet score	2.4 ± 0.9	2.3 ± 0.9	2.6 ± 0.9	<0.001
Vitamin and mineral supplementation, No. (%)	141,828 (34.8)	63,831 (22.9)	77,997 (60.5)	<0.001
Oily fish intake, No. (%)				<0.001
Never	43,939 (10.8)	34,584 (12.4)	9355 (7.3)	
<1 serving/week	136,400 (33.4)	99,277 (35.6)	37,123 (28.8)	
1 serving/week	154,621 (37.9)	101,230 (36.3)	53,391 (41.4)	
≥2 serving/week	73,063 (17.9)	44,089 (15.8)	28,974 (22.5)	
Nonoily fish intake, No. (%)				<0.001
Never	18,560 (4.5)	15,049 (5.4)	3511 (2.7)	
<1 serving/week	118,792 (29.1)	84,596 (30.3)	34,196 (26.5)	
1 serving/week	203,803 (49.9)	136,067 (48.7)	67,736 (52.6)	
≥2 serving/week	66,868 (16.4)	43,468 (15.6)	23,400 (18.2)	
Plasma Omega-3 PUFA, mmol/L	0.5 ± 0.2	0.5 ± 0.2	0.6 ± 0.2	<0.001
Plasma DHA, mmol/L	0.2 ± 0.1	0.2 ± 0.1	0.3 ± 0.1	<0.001
eGFR, mL/min/1.73 m^2^	91.7 ± 11.9	92.2 ± 12.1	90.5 ± 11.5	<0.001
UACR, mg/g	9.0 ± 5.7	8.8 ± 5.6	9.4 ± 5.8	<0.001

* Variables are presented as means ± SD or proportions. Abbreviation: BMI, body mass index; DHA, docosahexaenoic acid; eGFR, estimated glomerular filtration rate; PUFA, polyunsaturated fatty acid; TDI, Townsend Deprivation Index; UACR, urine albumin: creatinine ratio.

**Table 2:** The relationship between fish consumption with risk of incident chronic kidney disease.

Fish Consumption	Never	<1 Serving/Week	1 Serving/Week	≥2 Serving/Week	*p* for Trend	Per 1 Serving/Week
**Oily fish**						
No. of events	1227	3374	4122	2059		
Incidence rates *	2.4	2.1	2.3	2.4		
Adjusted model 1 ^†^	Ref	0.77 (0.72, 0.82)	0.71 (0.67, 0.76)	0.68 (0.64, 0.73)	<0.001	0.94 (0.92, 0.96)
Adjusted model 2 ^†^	Ref	0.90 (0.84, 0.96)	0.91 (0.85, 0.97)	0.86 (0.80, 0.93)	0.003	0.98 (0.96, 1.00)
Adjusted model 3 ^†^	Ref	0.88 (0.82, 0.94)	0.87 (0.82, 0.94)	0.83 (0.77, 0.90)	<0.001	0.97 (0.95, 0.99)
Adjusted model 4 ^†^	Ref	0.91 (0.84, 0.98)	0.90 (0.84, 0.97)	0.86 (0.79, 0.93)	0.002	0.97 (0.95, 0.99)
Adjusted model 5 ^†^	Ref	0.91 (0.85, 0.98)	0.90 (0.84, 0.97)	0.86 (0.79, 0.94)	0.002	0.97 (0.95, 0.99)
**Nonoily fish**						
No. of events	478	3036	5517	1751		
Incidence rates *	2.2	2.2	2.3	2.2		
Adjusted model 1 ^†^	Ref	0.84 (0.76, 0.93)	0.79 (0.72, 0.87)	0.78 (0.71, 0.87)	<0.001	0.97 (0.95, 0.99)
Adjusted model 2 ^†^	Ref	0.94 (0.85, 1.03)	0.94 (0.85, 1.03)	0.92 (0.83, 1.02)	0.281	0.99 (0.97, 1.01)
Adjusted model 3 ^†^	Ref	0.84 (0.76, 0.92)	0.83 (0.75, 0.91)	0.81 (0.72, 0.89)	0.004	0.98 (0.96, 1.00)
Adjusted model 4 ^†^	Ref	0.89 (0.80, 0.99)	0.88 (0.79, 0.98)	0.86 (0.77, 0.97)	0.065	0.98 (0.96, 1.00)
Adjusted model 5 ^†^	Ref	0.89 (0.80, 0.99)	0.88 (0.79, 0.98)	0.86 (0.77, 0.97)	0.057	0.98 (0.96, 1.00)

* Incidence rates were expressed as per 1000 person*–*years. ^†^ “Ref” means the reference group with an HR of 1.00; Adjusted Model 1: Adjusted for age, and sex; Adjusted Model 2: adjusted for the covariates in Model 1 and further adjusted for race, Townsend Deprivation Index, body mass index, smoking status, alcohol consumption, diabetes, hypertension, high cholesterol, vitamin or mineral supplementation, healthy diet score, and fish oil supplement; Adjusted Model 3: adjusted for the covariates in Model 2 and further adjusted for renal function (estimated glomerular filtration rate, and urine albumin: creatinine ratio); Adjusted Model 4: adjusted for the covariates in Model 3 and mutually adjusted for oily fish and nonoily fish consumption; Adjusted Model 5: adjusted for the covariates in Model 4 and further adjusted for CKD genetic risk scores.

**Table 3:** The relationship between circulating Omega-3 polyunsaturated fatty acid with risk of incident chronic kidney disease *.

	Quantiles of Omega-3 PUFA, mmol/L				*p* for Trend	Per SD Increment
	Q1	Q2	Q3	Q4		
**Omega-3 polyunsaturated fatty acid**						
Range	<0.37	0.37–<0.49	0.49–<0.64	≥0.64		
No. of events	632	657	680	614		
Incidence rates ^†^	2.2	2.3	2.4	2.1		
Adjusted model 1^‡^	Ref	0.89 (0.80, 0.99)	0.83 (0.74, 0.93)	0.67 (0.60, 0.76)	<0.001	0.87 (0.83, 0.90)
Adjusted model 2 ^‡^	Ref	0.90 (0.81, 1.01)	0.89 (0.80, 1.00)	0.79 (0.71, 0.89)	<0.001	0.93 (0.89, 0.97)
Adjusted model 3 ^‡^	Ref	0.91 (0.82, 1.02)	0.90 (0.81, 1.01)	0.83 (0.74, 0.94)	0.004	0.94 (0.90, 0.98)
Adjusted model 4 ^‡^	Ref	0.87 (0.78, 0.98)	0.83 (0.74, 0.94)	0.73 (0.63, 0.83)	<0.001	0.89 (0.84, 0.94)
Adjusted model 5 ^‡^	Ref	0.87 (0.78, 0.98)	0.82 (0.73, 0.93)	0.72 (0.63, 0.83)	<0.001	0.89 (0.84, 0.94)
**Docosahexaenoic acid**						
Range	<0.18	0.18–<0.22	0.22–<0.28	≥0.28		
No. of events	801	629	592	561		
Incidence rates ^†^	2.8	2.2	2.1	2		
Adjusted model 1 ^‡^	Ref	0.72 (0.65, 0.80)	0.62 (0.55, 0.69)	0.51 (0.46, 0.57)	<0.001	0.78 (0.74, 0.81)
Adjusted model 2 ^‡^	Ref	0.82 (0.74, 0.91)	0.76 (0.68, 0.85)	0.73 (0.64, 0.82)	<0.001	0.89 (0.85, 0.93)
Adjusted model 3 ^‡^	Ref	0.84 (0.76, 0.94)	0.80 (0.72, 0.90)	0.78 (0.69, 0.89)	<0.001	0.91 (0.87, 0.95)
Adjusted model 4 ^‡^	Ref	0.84 (0.76, 0.94)	0.80 (0.71, 0.89)	0.76 (0.67, 0.87)	<0.001	0.91 (0.87, 0.96)
Adjusted model 5 ^‡^	Ref	0.83 (0.75, 0.93)	0.79 (0.70, 0.89)	0.76 (0.67, 0.87)	<0.001	0.91 (0.87, 0.96)

* Analysis was performed in 98,223 participants with data on plasma fatty acid. ^†^ Incidence rates were expressed as per 1000 person–years. ^‡^ “Ref” means the reference group with an HR of 1.00; Adjusted Model 1: Adjusted for age, and sex; Adjusted Model 2: adjusted for the covariates in Model 1 and further adjusted for race, Townsend Deprivation Index, body mass index, smoking status, alcohol consumption, diabetes, hypertension, high cholesterol, vitamin or mineral supplementation, healthy diet score, and fish oil supplement; Adjusted Model 3: adjusted for the covariates in Model 2 and further adjusted for renal function (estimated glomerular filtration rate, and urine albumin: creatinine ratio); Adjusted Model 4: adjusted for the covariates in Model 3 and further adjusted for plasma saturated fatty acid, monounsaturated fatty acid, and omega-6 polyunsaturated fatty acid; Adjusted Model 5: adjusted for the covariates in Model 4 and further adjusted for CKD genetic risk score.

## Data Availability

The UK Biobank data are available on application to the UK Biobank, and the analytic methods and codes will be available from the corresponding authors on request.

## Supplementary Materials

The following supporting information can be downloaded at: https://www.mdpi.com/article/10.3390/nu15010022/s1, Figure S1: Flow chart of the participants in the current analysis; Table S1: Characteristics of the included (including 31519 participants with CKD at baseline) and excluded population in current study. Table S2: Disease definitions used in the UK Biobank study. Table S3: Baseline population characteristics by fish consumption. Table S4: Baseline population characteristics by quantiles of circulating Omega-3 polyunsaturated fatty acid.

## References

[1] Go A.S., Chertow G.M., Fan D., McCulloch C.E., Hsu C.Y. (2004). Chronic kidney disease and the risks of death, cardiovascular events, and hospitalization. N. Engl. J. Med..

[2] Lees J.S., Welsh C.E., Celis-Morales C.A., Mackay D., Lewsey J., Gray S.R., Lyall D.M., Cleland J.G., Gill J.M.R., Jhund P.S. (2019). Glomerular filtration rate by differing measures, albuminuria and prediction of cardiovascular disease, mortality and end-stage kidney disease. Nat. Med..

[3] Jha V., Garcia-Garcia G., Iseki K., Li Z., Naicker S., Plattner B., Saran R., Wang A.Y., Yang C.W. (2013). Chronic kidney disease: Global dimension and perspectives. Lancet.

[4] GBD Chronic Kidney Disease Collaboration (2020). Global, regional, and national burden of chronic kidney disease, 1990–2017: A systematic analysis for the Global Burden of Disease Study 2017. Lancet.

[5] Lee C.C., Adler A.I. (2012). Recent findings on the effects of marine-derived n-3 polyunsaturated fatty acids on urinary albumin excretion and renal function. Curr. Atheroscler. Rep..

[6] Noels H., Lehrke M., Vanholder R., Jankowski J. (2021). Lipoproteins and fatty acids in chronic kidney disease: Molecular and metabolic alterations. Nat. Rev. Nephrol..

[7] Shapiro H., Theilla M., Attal-Singer J., Singer P. (2011). Effects of polyunsaturated fatty acid consumption in diabetic nephropathy. Nat. Rev. Nephrol..

[8] Park I., Xun P., Tsinovoi C.L., Klemmer P., Liu K., He K. (2020). Intakes of long-chain omega-3 polyunsaturated fatty acids and non-fried fish in relation to incidence of chronic kidney disease in young adults: A 25-year follow-up. Eur. J. Nutr..

[9] Lauretani F., Semba R.D., Bandinelli S., Miller E.R., Ruggiero C., Cherubini A., Guralnik J.M., Ferrucci L. (2008). Plasma polyunsaturated fatty acids and the decline of renal function. Clin. Chem..

[10] Saglimbene V.M., Wong G., van Zwieten A., Palmer S.C., Ruospo M., Natale P., Campbell K., Teixeira-Pinto A., Craig J.C., Strippoli G.F.M. (2020). Effects of omega-3 polyunsaturated fatty acid intake in patients with chronic kidney disease: Systematic review and meta-analysis of randomized controlled trials. Clin. Nutr..

[11] Hoogeveen E.K., Geleijnse J.M., Kromhout D., Stijnen T., Gemen E.F., Kusters R., Giltay E.J. (2014). Effect of omega-3 fatty acids on kidney function after myocardial infarction: The Alpha Omega Trial. Clin. J. Am. Soc. Nephrol..

[12] de Boer I.H., Zelnick L.R., Ruzinski J., Friedenberg G., Duszlak J., Bubes V.Y., Hoofnagle A.N., Thadhani R., Glynn R.J., Buring J.E. (2019). Effect of Vitamin D and Omega-3 Fatty Acid Supplementation on Kidney Function in Patients with Type 2 Diabetes: A Randomized Clinical Trial. JAMA.

[13] Miller E.R., Juraschek S.P., Appel L.J., Madala M., Anderson C.A., Bleys J., Guallar E. (2009). The effect of n-3 long-chain polyunsaturated fatty acid supplementation on urine protein excretion and kidney function: Meta-analysis of clinical trials. Am. J. Clin. Nutr..

[14] Sherman R.E., Anderson S.A., Dal Pan G.J., Gray G.W., Gross T., Hunter N.L., LaVange L., Marinac-Dabic D., Marks P.W., Robb M.A. (2016). Real-World Evidence—What Is It and What Can It Tell Us?. N. Engl. J. Med..

[15] Sudlow C., Gallacher J., Allen N., Beral V., Burton P., Danesh J., Downey P., Elliott P., Green J., Landray M. (2015). UK biobank: An open access resource for identifying the causes of a wide range of complex diseases of middle and old age. PLoS Med..

[16] Collins R. (2012). What makes UK Biobank special?. Lancet.

[17] Bradbury K.E., Young H.J., Guo W., Key T.J. (2018). Dietary assessment in UK Biobank: An evaluation of the performance of the touchscreen dietary questionnaire. J. Nutr. Sci..

[18] Eastwood S.V., Mathur R., Atkinson M., Brophy S., Sudlow C., Flaig R., de Lusignan S., Allen N., Chaturvedi N. (2016). Algorithms for the Capture and Adjudication of Prevalent and Incident Diabetes in UK Biobank. PLoS ONE.

[19] Li X., Wang M., Song Y., Ma H., Zhou T., Liang Z., Qi L. (2021). Obesity and the relation between joint exposure to ambient air pollutants and incident type 2 diabetes: A cohort study in UK Biobank. PLoS Med..

[20] Levey A.S., Stevens L.A., Schmid C.H., Zhang Y.L., Castro A.F., Feldman H.I., Kusek J.W., Eggers P., Van Lente F., Greene T. (2009). A new equation to estimate glomerular filtration rate. Ann. Intern. Med..

[21] Zhang H., Wang B., Chen C., Sun Y., Chen J., Tan X., Xia F., Zhang J., Lu Y., Wang N. (2022). Sleep Patterns, Genetic Susceptibility, and Incident Chronic Kidney Disease: A Prospective Study of 370 671 Participants. Front. Neurosci..

[22] Kelly J.T., Su G., Zhang L., Qin X., Marshall S., González-Ortiz A., Clase C.M., Campbell K.L., Xu H., Carrero J.J. (2021). Modifiable Lifestyle Factors for Primary Prevention of CKD: A Systematic Review and Meta-Analysis. J. Am. Soc. Nephrol..

[23] Backes J., Anzalone D., Hilleman D., Catini J. (2016). The clinical relevance of omega-3 fatty acids in the management of hypertriglyceridemia. Lipids Health Dis..

[24] Wei M.Y., Jacobson T.A. (2011). Effects of eicosapentaenoic acid versus docosahexaenoic acid on serum lipids: A systematic review and meta-analysis. Curr. Atheroscler. Rep..

[25] Ebert T., Neytchev O., Witasp A., Kublickiene K., Stenvinkel P., Shiels P.G. (2021). Inflammation and Oxidative Stress in Chronic Kidney Disease and Dialysis Patients. Antioxid. Redox Signal..

[26] Djuricic I., Calder P.C. (2021). Beneficial Outcomes of Omega-6 and Omega-3 Polyunsaturated Fatty Acids on Human Health: An Update for 2021. Nutrients.

[27] Oppedisano F., Macrì R., Gliozzi M., Musolino V., Carresi C., Maiuolo J., Bosco F., Nucera S., Caterina Zito M., Guarnieri L. (2020). The Anti-Inflammatory and Antioxidant Properties of n-3 PUFAs: Their Role in Cardiovascular Protection. Biomedicines.

[28] Chang C.H., Tseng P.T., Chen N.Y., Lin P.C., Lin P.Y., Chang J.P., Kuo F.Y., Lin J., Wu M.C., Su K.P. (2018). Safety and tolerability of prescription omega-3 fatty acids: A systematic review and meta-analysis of randomized controlled trials. Prostaglandins Leukot. Essent. Fatty Acids.

[29] Fry A., Littlejohns T.J., Sudlow C., Doherty N., Adamska L., Sprosen T., Collins R., Allen N.E. (2017). Comparison of Sociodemographic and Health-Related Characteristics of UK Biobank Participants with Those of the General Population. Am. J. Epidemiol..

