# Supplementary material for: Habitual Fish Oil Supplementation and Incident Chronic Kidney Disease in the UK Biobank
Source: Nutrients. 2022 Dec 21;15(1):22. doi: 10.3390/nu15010022 (PMC9824577; doi:10.3390/nu15010022)
Supplement: Supplementary file 1 [file nutrients-15-00022-s001.zip › nutrients-2006454-supplementary.pdf]

Supplementary Online Content

Supplemental Figure S1. Flow chart of the participants in the current analysis.

Supplemental Table S1. Characteristics of the included (including 31519 participants with CKD at baseline) and excluded population in current study.

Supplemental Table S2. Disease definitions used in the UK Biobank study.

Supplemental Table S3. Baseline population characteristics by fish consumption**.**

Supplemental Table S4. Baseline population characteristics by quantiles of circulating Omega-3 polyunsaturated fatty acid.

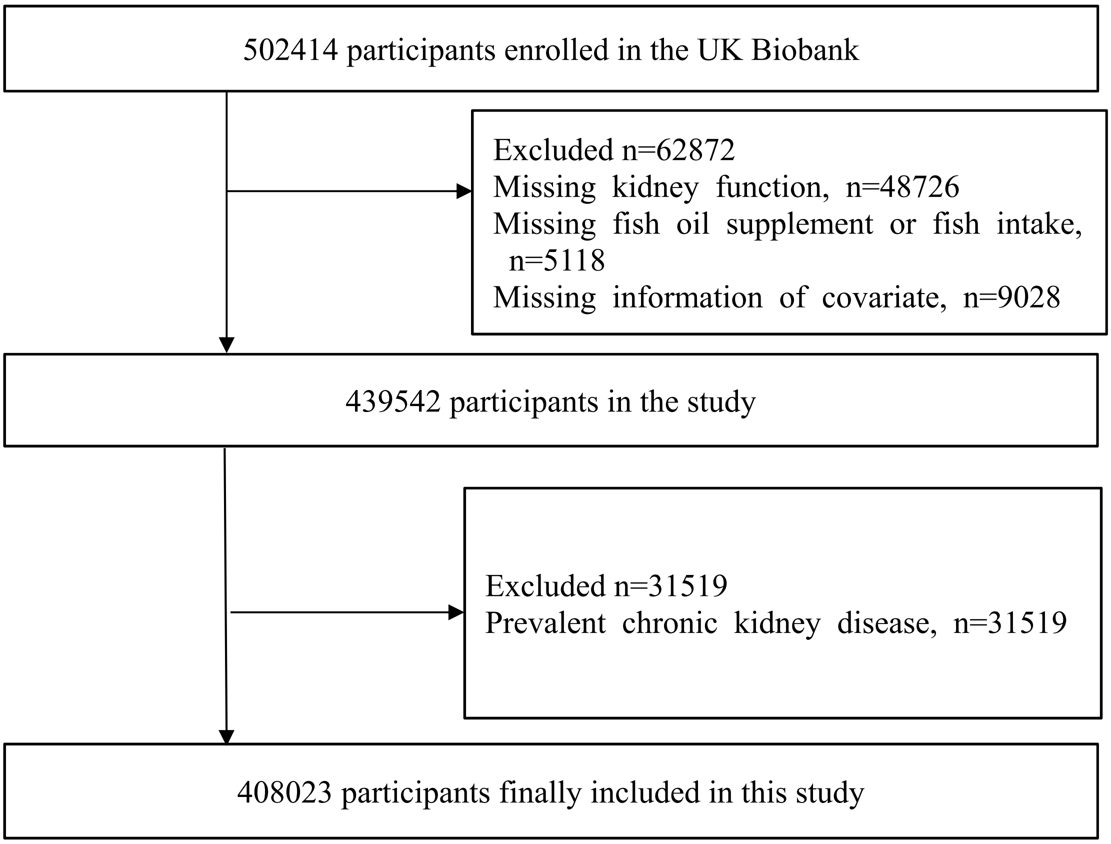

Supplement Figure S1. Flow chart of the participants in the current analysis.

Supplemental Table S1. Characteristics of the included (including 31519 participants with CKD at baseline) and excluded population in current study *.

	Excluded	Included
**N**	62872	439542
**Age, years**	56.4±8.3	56.5±8.1
**Male, No. (%)**	28249 (44.9)	200836 (45.7)
**White, No. (%)**	54163 (90.1)	418453 (95.2)
**TDI**	-0.7±3.4	-1.4±3
**BMI, kg/m^2**	27.9±5.2	27.4±4.7
**Smoking status, No. (%)**		
Never	32581 (54.4)	240897 (54.8)
Former	19720 (32.9)	153305 (34.9)
Current	7622 (12.7)	45340 (10.3)
**Alcohol consumption, No. (%)**		
Never	7138 (11.6)	33489 (7.6)
<1 times/week	15444 (25.2)	98394 (22.4)
1-2 times/week	15305 (24.9)	113965 (25.9)
3-4 times/week	12388 (20.2)	103036 (23.4)
>4 times/week	11096 (18.1)	90658 (20.6)
**Disease history, No. (%)**		
Diabetes	4096 (6.6)	21573 (4.9)
Hypertension	34954 (60.8)	243355 (55.4)
High cholesterol	11721 (21.6)	82085 (18.7)
**Healthy diet score**	2.4±0.9	2.4±0.9
**Vitamin and mineral supplementation, No. (%)**	19557 (35.2)	153134 (34.8)
**Fish oil supplementation, No. (%)**	16954 (29.9)	138845 (31.6)
**Oily fish intake, No. (%)**		
Never	7384 (12.6)	47440 (10.8)
<1 serving/week	18734 (31.9)	146125 (33.2)
1 serving/week	21853 (37.2)	166614 (37.9)
≥2 serving/week	10821 (18.4)	79363 (18.1)
**Nonoily fish intake, No. (%)**		
Never	3391 (5.7)	20097 (4.6)
<1 serving/week	17442 (29.5)	127382 (29)
1 serving/week	28516 (48.2)	219820 (50)
≥2 serving/week	9800 (16.6)	72243 (16.4)
**eGFR, mL/min/1.73 m^2**	91.3±15.2	90.8±13.3
**UACR, mg/g**	18.7±132.3	15.2±80.5

*Values are presented as means±SD or proportions.

Abbreviation: BMI, body mass index; CKD, chronic kidney disease; eGFR,

estimated glomerular filtration rate; TDI, Townsend Deprivation Index; UACR, urine albumin: creatinine ratio.

Supplemental Table S2. Disease definitions used in the UK Biobank study.

Disease	**ICD-9**	**ICD-10**	**OPCS-4**
Chronic kidney disease	585,5859	I12.0, I13.1, I13.2, N18.0, N18.3, N18.4, N18.5, N18.8, N18.9	M01

Abbreviations: ICD, International Classification of Diseases; OPCS, the Office of Population Censuses and Surveys Classification of Interventions and Procedures.

Supplemental Table S3. Baseline population characteristics by fish consumption*.

	Never	<1 serving/week	1 serving/week	≥2 serving/week	*P* value
Oily fish					
**N**	43939	136400	154621	73063	
**Age, years**	53.9±8.1	55.3±8	57.1±7.9	58.2±7.7	< 0.001
**Male, No. (%)**	20772 (47.3)	65463 (48)	68174 (44.1)	32522 (44.5)	< 0.001
**White, No. (%)**	41122 (93.6)	130461 (95.6)	148302 (95.9)	69097 (94.6)	< 0.001
**TDI**	-0.7±3.2	-1.4±3	-1.6±2.9	-1.3±3.1	< 0.001
**BMI, kg/m^2**	27.7±5.1	27.4±4.7	27.2±4.5	27.2±4.6	< 0.001
**Smoking status, No. (%)**					< 0.001
Never	24012 (54.6)	74978 (55)	86778 (56.1)	39371 (53.9)	
Former	13340 (30.4)	46418 (34)	54178 (35)	27229 (37.3)	
Current	6587 (15)	15004 (11)	13665 (8.8)	6463 (8.8)	
**Alcohol consumption, No. (%)**					< 0.001
Never	6166 (14)	9218 (6.8)	9285 (6)	5401 (7.4)	
<1 times/week	12544 (28.5)	31915 (23.4)	30915 (20)	14728 (20.2)	
1-2 times/week	11332 (25.8)	36076 (26.4)	40600 (26.3)	18294 (25)	
3-4 times/week	7703 (17.5)	31177 (22.9)	39721 (25.7)	18342 (25.1)	
>4 times/week	6194 (14.1)	28014 (20.5)	34100 (22.1)	16298 (22.3)	
**Disease history, No. (%)**					
Diabetes	2160 (4.9)	5346 (3.9)	6265 (4.1)	3490 (4.8)	< 0.001
Hypertension	22573 (51.4)	70287 (51.5)	84712 (54.8)	42287 (57.9)	< 0.001
High cholesterol	6723 (15.3)	20730 (15.2)	27695 (17.9)	15946 (21.8)	< 0.001
**Healthy diet score**	1.9±0.7	1.9±0.7	2.7±0.8	3.1±0.7	< 0.001
**Vitamin and mineral supplementation, No. (%)**	14464 (32.9)	41966 (30.8)	55192 (35.7)	30206 (41.3)	< 0.001
**Fish oil supplementation, No. (%)**	9355 (21.3)	37123 (27.2)	53391 (34.5)	28974 (39.7)	< 0.001
**eGFR, mL/min/1.73 m^2**	93.9±12.3	92.1±11.9	91.2±11.8	90.7±11.8	< 0.001
**UACR, mg/g**	8.8±5.7	8.6±5.5	9.1±5.7	9.4±5.8	< 0.001
Nonoily fish					
**N**	18560	118792	203803	66868	
**Age, years**	54±8.2	55.6±8	56.9±8	56.6±8.1	< 0.001
**Male, No. (%)**	8073 (43.5)	56098 (47.2)	93260 (45.8)	29500 (44.1)	< 0.001
**White, No. (%)**	16266 (87.6)	112419 (94.6)	196461 (96.4)	63836 (95.5)	< 0.001
**TDI**	-0.6±3.3	-1.3±3.1	-1.6±3	-1.4±3.1	< 0.001
**BMI, kg/m^2**	26.9±5	27.3±4.7	27.3±4.6	27.4±4.8	< 0.001
**Smoking status, No. (%)**					< 0.001
Never	10710 (57.7)	64999 (54.7)	112771 (55.3)	36659 (54.8)	
Former	5433 (29.3)	40577 (34.2)	71538 (35.1)	23617 (35.3)	

Current	2417 (13)	13216 (11.1)	19494 (9.6)	6592 (9.9)	
**Alcohol consumption, No. (%)**					< 0.001
Never	3490 (18.8)	8860 (7.5)	12717 (6.2)	5003 (7.5)	
<1 times/week	4966 (26.8)	28620 (24.1)	42412 (20.8)	14104 (21.1)	
1-2 times/week	4184 (22.5)	30705 (25.8)	54387 (26.7)	17026 (25.5)	
3-4 times/week	3149 (17)	26492 (22.3)	50777 (24.9)	16525 (24.7)	
>4 times/week	2771 (14.9)	24115 (20.3)	43510 (21.3)	14210 (21.3)	
**Disease history, No. (%)**					
Diabetes	929 (5)	4857 (4.1)	8462 (4.2)	3013 (4.5)	< 0.001
Hypertension	8868 (47.8)	61268 (51.6)	112320 (55.1)	37403 (55.9)	< 0.001
High cholesterol	2643 (14.2)	18952 (16)	36785 (18)	12714 (19)	< 0.001
**Healthy diet score**	2±0.7	1.9±0.8	2.5±0.9	2.9±0.7	< 0.001
**Vitamin and mineral supplementation, No. (%)**	7330 (39.5)	38202 (32.2)	70819 (34.7)	25477 (38.1)	< 0.001
**Fish oil supplementation, No. (%)**	3511 (18.9)	34196 (28.8)	67736 (33.2)	23400 (35)	< 0.001
**eGFR, mL/min/1.73 m^2**	95.8±12.1	92±12	91.2±11.8	91.6±11.9	< 0.001
**UACR, mg/g**	9.5±6	8.7±5.6	9±5.7	9.1±5.7	< 0.001

*Values are presented as means±SD or proportions.

Abbreviation: BMI, body mass index; eGFR, estimated glomerular filtration rate; TDI, Townsend Deprivation Index; UACR, urine albumin: creatinine ratio.

Supplemental Table S4. Baseline population characteristics by quantiles of circulating Omega-3 polyunsaturated fatty acid *.

	Q1	Q2	Q3	Q4	*P* value
Omega-3 polyunsaturated fatty acid					
**N**	24479	24478	24478	24479	
**Age, years**	53.8±8.4	55.7±8.1	57.1±7.7	58.6±7.2	< 0.001
**Male, No. (%)**	13438 (54.9)	11892 (48.6)	10743 (43.9)	8874 (36.3)	< 0.001
**White, No. (%)**	23208 (94.8)	23368 (95.5)	23445 (95.8)	23405 (95.6)	< 0.001
**TDI**	-1.1±3.2	-1.3±3.1	-1.5±3	-1.7±2.9	< 0.001
**BMI, kg/m^2**	27.2±5	27.5±4.7	27.4±4.6	27.2±4.3	< 0.001
**Smoking status, No. (%)**					< 0.001
Never	13358 (54.6)	13434 (54.9)	13454 (55)	13644 (55.7)	
Former	7559 (30.9)	8421 (34.4)	8864 (36.2)	9115 (37.2)	
Current	3562 (14.6)	2623 (10.7)	2160 (8.8)	1720 (7)	
**Alcohol consumption, No. (%)**					< 0.001
Never	2289 (9.4)	1771 (7.2)	1619 (6.6)	1539 (6.3)	
<1 times/week	6273 (25.6)	5523 (22.6)	5070 (20.7)	4725 (19.3)	
1-2 times/week	6692 (27.3)	6578 (26.9)	6302 (25.7)	5887 (24)	
3-4 times/week	5163 (21.1)	5831 (23.8)	6077 (24.8)	6300 (25.7)	
>4 times/week	4062 (16.6)	4775 (19.5)	5410 (22.1)	6028 (24.6)	
**Disease history, No. (%)**					
Diabetes	1206 (4.9)	1157 (4.7)	1056 (4.3)	839 (3.4)	< 0.001
Hypertension	11707 (47.8)	13074 (53.4)	13545 (55.3)	14324 (58.5)	< 0.001
High cholesterol	3266 (13.3)	4317 (17.6)	4693 (19.2)	4864 (19.9)	< 0.001
**Healthy diet score**	2.1±0.8	2.3±0.9	2.5±0.9	2.7±0.9	< 0.001
**Vitamin and mineral supplementation, No. (%)**	7010 (28.6)	7852 (32.1)	8898 (36.4)	10240 (41.8)	< 0.001
**Fish oil supplementation, No. (%)**	4441 (18.1)	6579 (26.9)	8599 (35.1)	11263 (46)	< 0.001
**eGFR, mL/min/1.73 m^2**	93.4±12.2	91.9±12	91.1±11.8	90.5±11.6	< 0.001
**UACR, mg/g**	8.4±5.5	8.7±5.6	9±5.7	9.7±5.9	< 0.001
Docosahexaenoic acid					
**N**	24472	24478	24480	24484	
**Age, years**	54.6±8.3	55.4±8.2	56.7±7.8	58.5±7.2	< 0.001
**Male, No. (%)**	15320 (62.6)	12079 (49.3)	9891 (40.4)	7657 (31.3)	< 0.001
**White, No. (%)**	23243 (95)	23382 (95.5)	23412 (95.6)	23389 (95.5)	< 0.001
**TDI**	-1±3.2	-1.3±3	-1.6±3	-1.8±2.9	< 0.001
**BMI, kg/m^2**	28.3±5.2	27.5±4.7	27±4.4	26.4±4.1	< 0.001
**Smoking status, No. (%)**					< 0.001
Never	12693 (51.9)	13402 (54.8)	13701 (56)	14094 (57.6)	
Former	7947 (32.5)	8369 (34.2)	8710 (35.6)	8933 (36.5)	
Current	3832 (15.7)	2707 (11.1)	2069 (8.5)	1457 (6)	

**Alcohol consumption, No. (%)**					< 0.001
Never	2440 (10)	1830 (7.5)	1528 (6.2)	1420 (5.8)	
<1 times/week	6674 (27.3)	5612 (22.9)	4860 (19.9)	4445 (18.2)	
1-2 times/week	6737 (27.5)	6629 (27.1)	6318 (25.8)	5775 (23.6)	
3-4 times/week	4898 (20)	5686 (23.2)	6211 (25.4)	6576 (26.9)	
>4 times/week	3723 (15.2)	4721 (19.3)	5563 (22.7)	6268 (25.6)	
**Disease history, No. (%)**					
Diabetes	1546 (6.3)	1121 (4.6)	942 (3.8)	649 (2.7)	< 0.001
Hypertension	13233 (54.1)	13060 (53.4)	12914 (52.8)	13443 (54.9)	< 0.001
High cholesterol	4159 (17)	4336 (17.7)	4463 (18.2)	4182 (17.1)	< 0.001
**Healthy diet score**	2.1±0.8	2.3±0.9	2.5±0.9	2.8±0.9	< 0.001
**Vitamin and mineral supplementation, No. (%)**	6796 (27.8)	7727 (31.6)	8902 (36.4)	10575 (43.2)	< 0.001
**Fish oil supplementation, No. (%)**	4464 (18.2)	6339 (25.9)	8519 (34.8)	11560 (47.2)	< 0.001
**eGFR, mL/min/1.73 m^2**	92.7±12.4	92.1±12	91.4±11.8	90.7±11.5	< 0.001
**UACR, mg/g**	8.1±5.5	8.6±5.5	9.2±5.7	9.9±5.9	< 0.001

*Values are presented as means±SD or proportions.

Abbreviation: BMI, body mass index; eGFR, estimated glomerular filtration rate; TDI, Townsend Deprivation Index; UACR, urine albumin: creatinine ratio.
